# Neighborhood Influences on Perceived Social Support Among Parents: Findings from the Project on Human Development in Chicago Neighborhoods

**DOI:** 10.1371/journal.pone.0034235

**Published:** 2012-04-06

**Authors:** Shalini A. Tendulkar, Karestan C. Koenen, Erin C. Dunn, Stephen Buka, S. V. Subramanian

**Affiliations:** 1 Institute for Community Health, Harvard Medical School, Cambridge Health Alliance, Cambridge, Massachusetts, United States of America; 2 Department of Epidemiology, Mailman School of Public Health, Columbia University, New York, New York, United States of America; 3 Department of Society, Human Development and Health, Harvard School of Public Health, Boston, Massachusetts, United States of America; 4 Department of Community Health, Brown University, Providence, Rhode Island, United States of America; 5 Department of Society, Human Development and Health, Harvard School of Public Health, Boston, Massachusetts, United States of America; Hungarian Academy of Sciences, Hungary

## Abstract

**Background:**

Social support is frequently linked to positive parenting behavior. Similarly, studies increasingly show a link between neighborhood residential environment and positive parenting behavior. However, less is known about how the residential environment influences parental social support. To address this gap, we examine the relationship between neighborhood concentrated disadvantage and collective efficacy and the *level* and *change* in parental caregiver perceptions of non-familial social support.

**Methodology/Principal Findings:**

The data for this study came from three data sources, the Project on Human Development in Chicago Neighborhoods (PHDCN) Study's Longitudinal Cohort Survey of caregivers and their offspring, a Community Survey of adult residents in these same neighborhoods and the 1990 Census. Social support is measured at Wave 1 and Wave 3 and neighborhood characteristics are measured at Wave 1. Multilevel linear regression models are fit. The results show that neighborhood collective efficacy is a significant (ß = .04; SE = .02; p = .03), predictor of the positive *change* in perceived social support over a 7 year period, however, not of the *level* of social support, adjusting for key compositional variables and neighborhood concentrated disadvantage. In contrast concentrated neighborhood disadvantage is not a significant predictor of either the level or change in social support.

**Conclusion:**

Our finding suggests that neighborhood collective efficacy may be important for inducing the perception of support from friends in parental caregivers over time.

## Introduction

Social support, defined as “…information leading the subject to believe that he is cared for and loved, esteemed, and a member of a network of mutual obligations” has been consistently linked to psychological and physical health across numerous studies [1]–[3]. Perceived social support among parents is also deemed to be an important determinant of quality parenting, namely parental responsiveness to a child's needs [4], lower risk of parent-to-child physical aggression [5], and increased parental effectiveness [6]. In contrast, inadequate parental support or social isolation has been linked to diminished parental well-being, mental health problems, and damaging parenting practices [7]–[11]. The residential context, or neighborhood environment, has been identified as an important determinant of both positive and negative parenting [12]–[15]. For example, in a study of mothers and their 3-year old children, Klebanov et al. (1994) found that neighborhood poverty was negatively and associated with maternal warmth, even after adjusting for salient family variables such as family poverty.

Despite the accumulation of evidence on the importance of parental perceived social support and the residential environment for parenting, only a few studies have examined the effect of the residential environment itself on parental perceived social support. Although small in number, these studies suggest that the residential or neighborhood environment shapes parent's perceived social support. For example, in a study of single African-American mothers, Ceballo and McLoyd (2002) found that for mothers, living in a disordered community environment characterized by low maternal neighborhood ratings, high violent crime rates and a high percentage of families living in poverty, the potential positive effect of parental social support, on nurturing parenting behaviors was significantly attenuated [16]. In another ecological study of neighborhood socioeconomic status (SES) and child maltreatment rates, Garbarino and Sherman (1980) found higher child maltreatment rates in neighborhoods with fewer economic resources [9]. They concluded this finding was explained by diminished access to social resources in the neighborhood.

While these studies suggest that parental perceived social support may be contextually patterned, or influenced by features of the neighborhood environment, there is insufficient empirical evidence to demonstrate a conclusive relationship. In particular, it is unclear whether social ties (i.e. social relationships) are more salient than the “activation” of these social ties to engage in collective action, a concept termed neighborhood collective efficacy. Collective efficacy is comprised of two related constructs: mutual trust (referred to as social cohesion) and the willingness of neighborhoods to intervene for the public good (referred to as informal social control) [17]. Collective efficacy has been examined in relation to outcomes such as partner violence [18], self-rated health [19] and adolescent suicide [20].

Studies have examined the two sub-components of collective efficacy – neighborhood level informal social control and social cohesion – independently in relation to family functioning. For example, Kohen et al (2008) found associations between residing in neighborhoods with low social cohesion and maternal depression and family dysfunction. Similarly, in another study, researchers reported that an increase in neighborhood informal social control was associated with a decrease in neglectful and psychologically harsh parenting [15] However, we found only one study that examined neighborhood collective efficacy in relation to parenting [21]. Simon et al (2005) found increases in the authoritative parenting of African American caregivers, in neighborhoods with increasing collective efficacy. However we found no studies which examine collective efficacy in relation to parental perceived social support though we hypothesize that these phenomena are related.

While few studies examine neighborhood characteristics in the context of parental perceived social support, numerous studies link neighborhood economic deprivation to a wide range of negative health and mental health related outcomes [22]–[26]. It is believed that neighborhood social ties and interactions as well as norms and collective efficacy represent two important pathways through which economic deprivation impact individual outcomes. For example, parents who reside in economically deprived neighborhoods may have limited access to social resources and feel more socially isolated, making them less likely to engage in collective social action [27], [28]. In contrast, parents living in resource rich environments characterized by mutual trust and the potential for collective action, may be more likely to share parenting strategies, resources and feedback and ultimately feel more supported.

Given the current gaps in the literature and the theoretical relationships described above, we use data from a longitudinal study of parents to explore three research questions: First, to examine whether there is an association between neighborhood concentrated disadvantage and collective efficacy and the *level* of perceived social support parents report from friends at one point in time (cross-sectional analysis), second to examine whether neighborhood concentrated disadvantage and collective efficacy induce *changes* in perceived social support over time (longitudinal analysis) and third to examine whether there is an interaction between neighborhood concentrated disadvantage and collective efficacy in both the cross-sectional and longitudinal analysis. We focus on both cross-sectional and longitudinal analyses, given that social support can fluctuate over the lifespan [29], [30].

## Methods

### Ethics Statement

The Harvard School of Public Health Human Subjects Committee determined that this research was exempt (Protocol #P14989-101)

### Study Design

Data for these analyses came from the Project on Human Development in Chicago Neighborhoods (PHDCN), a landmark study conducted from 1994 to 2001 investigating the individual, family, and neighborhood-level causes and consequences of youth exposure to urban violence [31]. The PHDCN consisted of two main components: (1) a *community survey* (CS), collected from 1994–1995 of residents living in urban neighborhoods which was aimed at understanding the social, economic, organizational, political, and cultural structures and processes of those neighborhoods [32] and (2) a *longitudinal cohort survey* (LCS) of children, adolescents, and young adults ages 0, 3, 6, 9, 12, 15 and 18 living in those neighborhoods collected from 1994–1997 (Wave 1), 1997–1999 (Wave 2), and 2000–2001 (Wave 3). To obtain more information about the structural (i.e. socio-demographic) characteristics of each neighborhood, PHDCN investigators also linked these two data sources to the 1990 US Census [33]. For this study, we utilized the 1990 Census measure given its proximity to the Wave 1 data collection, started in 1994. In order to be consistent, we also utilized the 1990 census data in the longitudinal analysis.As described below, we used data from the LCS to construct the parent-level variables; data from the CS and Census were used to derive the neighborhood-level variables.

#### Community Survey (CS)

To obtain a sample of participants for both the CS and LCS, PHDCN investigators began by dividing the city of Chicago into 847 populated census tracts, which were then collapsed to form 343 ecologically meaningful, geographically compact, homogenous neighborhood clusters (NCs). The neighborhood clusters were approximately 8000 people large and were homogenous on key census indicators. This was in contrast to the 77 communities in Chicago, which consisted of approximately 40,000 people each and were less likely to represent “true” neighborhoods. For the CS, investigators used a three-stage cluster sampling design. At the first stage, city blocks were randomly sampled within each of the 343 NCs. At the second stage, dwelling units were randomly sampled within each city block. In most cases, all dwelling units in a NC were selected, though in large NCs, census blocks were sampled using probability proportional to size sampling methods. At the third stage, one adult resident (aged 18 and over) within each dwelling unit was randomly selected and interviewed for the CS. CS respondents ranged in age from 18 to 83, were predominately female (65%), and representative of the neighborhoods from which the LCS sample was drawn. While residents were interviewed from all areas of Chicago, a greater percentage of respondents were included in the CS that represented the NCs of the LCS.

#### Longitudinal Cohort Survey (LCS)

To obtain a sample of participants for the LCS, investigators stratified the 343 NCs into 7 levels of race/ethnicity and 3 levels of socioeconomic status (SES), resulting in 21 strata and 80 NCs (note: three strata did not contain any NCs). A list of all dwelling units in the 80 NCs was enumerated, and probability proportional to size sampling methods were used to select blocks, dwelling units, and persons within dwelling units. Households with children (and pregnancies) within 6 months of the target cohort age (0, 3, 6, 9, 12, 15 and 18) were selected to participate in the LCS. All household members were invited to participate in the study, which followed parents (i.e. individuals who spent the most time caring for the child and resided with the child at least 5 nights per week) and children ages 0, 3, 6, 9, 12, 15 and 18 over a period of 7 years (from 1994 to 2001) across three waves of data collection.

For all LCS cohorts except 0 and 18, both parents and children were interviewed. Separate research assistants administered the parent and child interviews. Interviews took place primarily in-person, though participants who declined to complete in-person interviews were interviewed via phone. Interviews were conducted in Spanish, English, and Polish and interpreters were provided for participants who spoke other languages. Participants were compensated between $5–$20 per interview depending on their age and the wave of data collection. Child participants were interviewed on a range of topics including language development, substance use, values, and sensation-seeking traits, while parents were interviewed on topics including family structure, parent-child relationships, and family mental health.

Of 8,304 eligible participants, 6226 were interviewed at Wave 1 (75% completion) [34]. Wave 1 data collection occurred between 1994 and 1997. Wave 2 data were collected from 1997 to 1999 (85.9% completion) and Wave 3 data collection occurred from 2000–2001 (78.19% completion). Data from the Wave 1 and Wave 3 LCS were used in this study. The PHDCN data presents a unique opportunity to examine a longitudinal cohort of children and families and assess neighborhoods from an independent sample of neighborhood residents from urban Chicago.

### Full and Analytic Samples

At Wave 1, 6226 parents of children in cohorts 0, 3, 6, 9, 12, 15 and 18 completed an interview for the LCS. Therefore the original dataset consisted of 6226 rows of data on children ages 0, 3, 6, 9, 12, 15 and 18 years old (i.e. 1 child per row). Parental caregivers of 18 year olds were not eligible for inclusion in this study mainly because they were not asked the outcome of interest. Multiple children could be nested within a caregiver but since the dataset was at the child level and we were interested in caregiver level data, we selected one row per parental caregiver (i.e. one child row) to use in our analysis. All the variables used in the analysis were caregiver level variables, which were identical across all children in the same family (e.g. all children with the same parental caregiver, had the same educational level recorded for that caregiver). We also noted that some caregivers changed from Wave 1 to Wave 3 and therefore we excluded them from our analysis. Technically nobody was excluded from the analytic sample size through these steps therefore we did not implement a sensitivity analysis. At this stage our analytic sample size was 2782. We did exclude approximately 3% of parents from the model analysis as these parents did not have complete data on the outcome of interest at Wave 1 and Wave 3. As this number was less than 5% of the overall sample we did not implement a sensitivity analysis.

Finally, we only included parents with complete parental perceived social support data (i.e. parents with no missing responses on the 7 items). We choose to include participants with complete data, rather than impute in order to be conservative in our estimates.

### Measures

The variables in this study came from measures included in the CS, LCS, or Census and tapped information about parents and the neighborhood environment. These measures are described below.

#### Perceived Social Support

Perceived social support from friends and family members was assessed among parents using a 20 item Provision of Social Relations (PSRP) instrument (Table 1), which queries parents about the social support they receive from family members and friends [35]. For this study, we examined perceived support from friends only, given our hypothesis that this type of social support would be strongly related to the neighborhood environment. Support from friends was measured on a Likert-type scale (response options were 1 = very true; 2 = somewhat true; 3 = not true) with eight items measured at both Wave 1 and Wave 3. The PSRP was not implemented at Wave 2, therefore we were restricted to analyzing this measure at two waves. The items comprising this scale demonstrate good internal consistency reliability in this sample (α = 0.75).

**Table 1:** Items Measuring Caregiver Perceived Friend Social Support.

When I'm with my friends I feel completely able to relax and be myself.
I share the same approach to life that many of my friends do.
People who know me trust me and respect me.
When I want to go out to do things, I know that many of my friends would enjoy doing these things with me.
I have at least one friend that I could tell anything to.
I feel very close to some of my friends
People who know me think I am good at what I do.
My friends would take the time to talk about my problems, should I ever want to.
Even when I am with my friends, I feel alone.^A^

A Items not included in Wave 3 social support measure, therefore excluded from construction of final measure.

We used these data to create several different representations of perceived social support among parents. First, we derived a mean social support score for each parental caregiver, based on the average response to the eight social support items at each wave. Second, we constructed two representations of this mean score for each set of analyses. For the cross-sectional analysis, we utilized the mean score for each caregiver at Wave 1. For the longitudinal analysis, we calculated a difference score (Wave 3 mean minus Wave 1 mean) for each parent caregiver. We constructed a difference score rather than controlling for baseline social support, based on evidence that controlling for the baseline score of a predictor in a longitudinal model can result in spuriously inflated coefficients [36].

#### Neighborhood Features

Measures of the neighborhood environment tapped both structural or socio-demographic measures of communities and social characteristics or attributes of social relationships of each neighborhood and came from either the 1990 Census or the 1995 CS data. We used these sources to construct two measures of neighborhood environment. The first was neighborhood concentrated disadvantage, which was derived by PHDCN investigators using a factor analysis from variables collected in the 1990 Census [27]. This factor included items that corresponded to percent below the poverty line, percent on public assistance, percent unemployed, percent female-headed households, percent under age 18, and percent African American.

The second measure, neighborhood collective efficacy, was constructed from LCS caregiver reports. In the LCS, respondents were asked to indicate their level of agreement with twelve statements; six items measured neighborhood *informal social control* or willingness to intervene on behalf of the common good or (i.e. if children were spray-painting graffiti on a local building) and six items measured *social cohesion*, or connectedness between neighbors (i.e. this is a close knit community). To generate neighborhood-level values for these variables, we calculated a mean neighborhood score across the 12 items. We grand-mean centered all neighborhood measures to facilitate interpretability. This procedure allows us to interpret the outcome for a neighborhood with the average level of each neighborhood measure.

#### Covariates

Throughout our analyses, we controlled for covariates obtained via parental self-report at Wave 1 of the LCS. These included: age (continuous), race/ethnicity (0 = White,1 = Black, 2 = Hispanic, 3 = Other), education (0 = less than high school, 1 = high school, 2 = greater than high school), sex (0 = female, 1 = male), household salary (0 = more than $50,000, 1 = between $40,000–$49,999, 2 = $30,000–$39,999, 3 = $20,000–$29,999, 4 = $10,000–$19,999, 5 = $0–$9,999), and marital status (0 = married, 1 = single, 2 = partnered). Given that we imputed household salary information for approximately 4% of cases, we included an imputation indicator.

### Analytic Approach

We began by conducting univariate and bivariate analyses to examine sample demographic characteristics, the distribution of and interrelationships between the predictors and outcomes, and evaluate the extent of missing data present. We then utilized multi-level linear regression for the cross-sectional analysis (i.e. examine the association between neighborhood concentrated disadvantage and collective efficacy on mean levels of social support at Wave 1) and longitudinal analysis (i.e. examine the association between neighborhood concentrated disadvantage and collective efficacy on the change in social support from Wave 1 to Wave 3). For both the cross-sectional and longitudinal analyses, we constructed all regression models in a sequential fashion. Specifically, we began by fitting a null or intercept-only model. We then introduced the covariates into the model, individually. First we examined neighborhood concentrated disadvantage and collective efficacy, individually in models with the demographic variables. Second, we examined the two neighborhood variables in the model simultaneously. And finally, we introduced an interaction term of the two neighborhood variables. We reported parameter estimates and standard errors, and variance estimates for each model. We performed all analyses in SAS 9.1. All models were estimated using SAS PROC MIXED.

## Results

### Sample Demographics

Table 2 shows the demographic characteristics of this sample at Wave 1. The sample was predominantly female (95%), Hispanic (42%) and included 47% of parents had less than a high school education. Approximately 43% of the sample reported earning less than 20,000 dollars a year. The majority of the sample was married (56%) and the mean age was 33 years.

**Table 2:** Sociodemographic Characteristics of Parental Caregivers and Mean Scores, Standard Deviations and Correlations Focusing on the Level of and Change in Perceived Social Support Scale Among Parental Caregivers (n = 2782).

		n (%)/Mean (SD)	Wave 1 Social Support; Mean (SD)	P-value	Change in Social Support from Wave 1 to Wave 3; Mean (SD)	P-value
**Caregiver Category**	**Sub-Category**					
*Gender*	Female	2636 (94.75)	1.57 (.39)	0.25	0.07 (0.41)	0.01
	Male	127 (4.65)	1.55 (.36)		0.09(0.35)	
	Missing	19 (.68)				
*Race/Ethnicity*	White	536 (19.27)	1.73 (0.29)	<.0001	0.05 (0.30)	0.52
	Hispanic	1181 (42.45)	1.47 (0.43)		0.09 (0.48)	
	Black	900 (32.35)	1.60 (0.34)		0.07 (0.37)	
	Other	117 (4.21)	1.61 (0.35)		0.06 (0.38)	
	Missing	48 (1.73)	1.61 (0.36)		0.09 (0.34)	
*Education*	>HS	1313 (47.20)	1.66 (0.33)	<.0001	0.07 (0.34)	0.70
	HS	361 (12.98)	1.55 (0.38)		0.09 (0.40)	
	<HS	1038(37.31)	1.46 (0.42)		0.08 (0.49)	
	Missing	70 (2.52)	1.45 (0.43)		−0.03 (0.54)	
*Household Salary*	>$50,000	482 (17.33)	1.73 (0.30)	<.0001	0.07 (0.29)	0.96
	$40,000–$49,999	243 (8.73)	1.66 (0.32)		0.09 (0.32)	
	$30,000–$39,999	344 (12.37)	1.60 (0.37)		0.09 (0.40)	
	$20,000–$29,999	494 (17.76)	1.53 (0.39)		0.07 (0.43)	
	$10,000–$19,999	537 (19.30)	1.50 (0.41)		0.07 (0.46)	
	<$9,999	664 (23.87)	1.48 (0.40)		0.07 (0.47)	
	Missing	18 (.65)	-		-	
*Marital Status*	Married	1539 (55.32)	1.59 (0.39)	<.0001	0.06 (0.41)	0.12
	Single	855 (30.73)	1.56 (0.36)		0.08 (0.39)	
	Partnered	359 (12.90)	1.47 (0.41)		0.11 (0.47)	
	Missing	29 (1.04)	1.44 (0.27)		0.25 (0.18)	
*Caregiver Age (Mean SD)*		32.90 (8.41)	0.12***		.12***	
*Salary Imputation Indicator*	Yes	112 (4.03)	1.57 (0.38)	0.00	0.13 (0.48)	0.02
	No	2652 (95.33)	1.47 (0.47)		0.07 (0.41)	
	Missing	18 (.65)	-		-	
*W1 Friend Social Support (Mean SD)*		1.57 (0.39)	-	-	-	-
*W3 Friend Social Support (Mean SD)*		1.64 (0.37)	-	-	-	-

### Means and Correlations

Mean comparison analyses were conducted to determine associations between the demographic variables and both the *level* and *change* in caregiver perceived social support (See Table 2). No significant gender differences in the *level* of social support were observed. However, white caregivers as compared with all caregivers in other racial/ethnic groups (p<.0001), caregivers with more than a high school education as compared with less educated caregivers (p<.0001), caregivers with a household salary greater than 50 K (p<.0001) and married caregivers, as compared with caregivers with all other marital status types (p<.0001) had a significantly higher level of social support at Wave 1. In contrast, there were no significant associations between any of the demographic variables and *change* in social support, with the exception of gender (p = .0106).

### Cross-Sectional Analyses Focusing on Mean Levels of Social Support

In Table 3 we present the results from multi-level linear regression models examining the adjusted associations between the neighborhood variables and parental caregiver mean levels of social support. The null model (not shown in table) describes the mean level of social support in this sample of parental caregivers (ß = 1.57; SE = .01; p = <.001). In a model that adjusted for covariates, neighborhood concentrated disadvantage and collective efficacy were not significantly associated with caregiver level of social support. As noted in Table 4, while 1.2% of the variation in parental caregiver perceived social support at Wave 1 was attributable to neighborhood variations in social support, this effect was rendered null when compositional (i.e. caregiver) characteristics were introduced into the model.

**Table 3:** Multilevel OLS Regression Coefficients, Standard Errors and P-values for Models of Effects of Caregiver and Neighborhood Characteristics on the *Level* of Parental Support.

		Model 1			Model 2			Model 3			Model 4			Model 5		
Caregiver Category	Sub-Category	Est	SE	P	Est	SE	P	Est	SE	P	Est	SE	P	Est	SE	P
*Intercept*		1.79	0.02	<.0001	1.79	0.02	<.0001	1.78	0.02	<.0001	1.78	0.02	<.0001	1.78	0.03	<.0001
*Gender*	Female (ref)															
	Male	−0.10	0.03	0.00	−0.10	0.03	0.00	−0.10	0.03	0.00	−0.10	0.03	0.00	−0.10	0.03	0.00
*Race/Ethnicity*	White (ref)															
	Hispanic	−0.06	0.02	0.00	−0.06	0.03	0.02	−0.06	0.02	0.02	−0.06	0.03	0.02	−0.06	0.03	0.03
	Black	−0.16	0.02	<.0001	−0.15	0.02	<.0001	−0.15	0.02	<.0001	−0.15	0.02	<.0001	−0.15	0.02	<.0001
	Other	−0.07	0.04	0.06	−0.07	0.04	0.06	−0.07	0.04	0.07	−0.07	0.04	0.07	−0.07	0.04	0.07
*Education*	>HS (ref)															
	HS	−0.07	0.02	0.00	−0.07	0.02	0.00	−0.07	0.02	0.00	−0.07	0.02	0.00	−0.07	0.02	0.00
	<HS	−0.10	0.02	<.0001	−0.10	0.02	<.0001	−0.10	0.02	<.0001	−0.10	0.02	<.0001	−0.10	0.02	<.0001
*Household Salary*	>$50,000 (ref)															
	$40,000–$49,999	−0.01	0.03	0.64	−0.01	0.03	0.66	−0.01	0.03	0.70	−0.01	0.03	0.69	−0.01	0.03	0.69
	$30,000–$39,999	−0.05	0.03	0.06	−0.05	0.03	0.07	−0.05	0.03	0.09	−0.05	0.03	0.09	−0.05	0.03	0.09
	$20,000–$29,999	−0.10	0.03	0.00	−0.10	0.03	0.00	−0.09	0.03	0.00	−0.09	0.03	0.00	−0.09	0.03	0.00
	$10,000–$19,999	−0.11	0.03	<.0001	−0.11	0.03	<.0001	−0.10	0.03	0.00	−0.10	0.03	0.00	−0.10	0.03	0.00
	$0–$9,999	−0.13	0.03	<.0001	−0.13	0.03	<.0001	−0.13	0.03	<.0001	−0.13	0.03	<.0001	−0.13	0.03	<.0001
*Marital Status*	Married (ref)															
	Single	0.01	0.02	0.58	0.01	0.02	0.57	0.01	0.02	0.59	0.01	0.02	0.60	0.01	0.02	0.60
	Partnered	−0.05	0.02	0.02	−0.05	0.02	0.03	−0.05	0.02	0.03	−0.05	0.02	0.03	−0.05	0.02	0.03
*Caregiver Age*		0.00	0.00	0.00	0.00	0.00	0.00	0.00	0.00	0.00	0.00	0.00	0.00	0.00	0.00	0.00
*Salary Imputation*	No (ref)															
	Yes	−0.05	0.04	0.16	−0.05	0.04	0.16	−0.05	0.04	0.16	−0.05	0.04	0.16	−0.05	0.04	0.16
*Neighborhood*																
Disadvantage (CD)					−0.01	0.01	0.69				0.00	0.01	0.89	0.00	0.01	0.92
Collective Efficacy (CE)								0.02	0.01	0.18	0.02	0.01	0.20	0.02	0.02	0.25
CD*CE														0.00	0.02	0.93

**Table 4:** Variance Parameter Estimates for Baseline Parental Caregiver Perceived Social Support.

	NeighborhoodLevelEst(SE)	CaregiverLevelEst(SE)	ICC^A^
**Null Model**	0.0017(0.0012)***	0.1422 (0.0039)***	1.2%
**Adjusted for compositional (i.e. caregiver) characteristics**	-	0.132(0.0037)***	-
**Adjusted for neighborhood concentrated disadvantage**	-	0.132(0.0037)***	-
**Adjusted for neighborhood collective efficacy**	-	0.132(0.0037)***	-

A Intra-class correlation coefficient, proportion of the unexplained variation in parental caregiver perceived social support attributable to the neighborhood level.

∼ p<.10,

* p<.05,

** p<.01,

*** p<.001.

### Longitudinal Analyses Focusing on Change in Social Support

Table 5 presents the results from the multi-level linear regression models examining the adjusted associations between the neighborhood variables and change in the caregiver level of social support. The null model (not shown in table) describes the mean change in social support in this sample of parental caregivers (ß = .07; SE = .07; p = <.001). After controlling for caregiver covariates neighborhood collective efficacy was positively and significantly associated with the changes in a caregiver's perceived social support (ß = .04; SE = .02; p = .03), such that each one unit change in neighborhood collective efficacy was associated with .04 increase in caregiver perceived social support from Wave 1 to Wave 3. However, neighborhood concentrated disadvantage was not significantly associated with the change in caregiver perceived social support. The interaction between neighborhood collective efficacy and concentrated disadvantage was also not significant. Finally, as noted in Table 6, while .1% of the variation in the change in parental caregiver perceived social support was attributable to neighborhood variations in social support, this effect was rendered null when compositional (i.e. caregiver) characteristics were introduced into the model.

**Table 5:** Multilevel OLS Regression Coefficients, Standard Errors and P-values for Models of Effects of Caregiver and Neighborhood Characteristics on the *Change* in Parental Support.

		Model 1			Model 2			Model 3			Model 4			Model 5		
		Est	SE	P	Est	SE	P	Est	SE	P	Est	SE	P	Est	SE	P
*Intercept*		0.06	0.02	0.01	0.04	0.03	0.11	0.04	0.02	0.14	0.03	0.03	0.25	0.03	0.03	0.34
*Gender*	Female (ref)															
	Male	0.06	0.04	0.10	0.06	0.04	0.11	0.07	0.04	0.10	0.06	0.04	0.10	0.06	0.04	0.10
*Race/Ethnicity*	White (ref)															
	Hispanic	0.01	0.03	0.79	0.03	0.03	0.35	0.03	0.03	0.34	0.03	0.03	0.27	0.03	0.03	0.26
	Black	0.03	0.03	0.19	0.04	0.03	0.10	0.06	0.03	0.04	0.06	0.03	0.03	0.06	0.03	0.04
	Other	0.01	0.04	0.85	0.02	0.04	0.73	0.02	0.04	0.66	0.02	0.04	0.63	0.02	0.04	0.62
*Education*	>HS (ref)															
	HS	0.01	0.03	0.60	0.01	0.03	0.56	0.01	0.03	0.65	0.01	0.03	0.63	0.01	0.03	0.63
	<HS	0.00	0.02	0.82	0.00	0.02	0.96	0.00	0.02	0.92	0.00	0.02	0.96	0.00	0.02	0.96
*Salary*	>$50,000 (ref)															
	$40,000–$49,999	0.03	0.02	0.14	0.01	0.03	0.85	0.01	0.03	0.83	0.01	0.03	0.81	0.01	0.03	0.80
	$30,000–$39,999	0.04	0.03	0.15	−0.01	0.03	0.87	0.00	0.03	0.95	0.00	0.03	0.98	0.00	0.03	0.99
	$20,000–$29,999	0.00	0.03	0.92	−0.02	0.03	0.45	−0.02	0.03	0.54	−0.02	0.03	0.58	−0.02	0.03	0.59
	$10,000–$19,999	−0.01	0.03	0.75	−0.03	0.03	0.30	−0.03	0.03	0.40	−0.02	0.03	0.44	−0.02	0.03	0.45
	$0–$9,999	−0.03	0.03	0.32	−0.04	0.03	0.19	−0.04	0.03	0.26	−0.03	0.03	0.29	−0.03	0.03	0.30
*Marital Status*	Married (ref)															
	Single	−0.04	0.03	0.19	0.03	0.02	0.12	0.03	0.02	0.15	0.03	0.02	0.14	0.03	0.02	0.14
	Partnered	−0.05	0.03	0.10	0.04	0.03	0.11	0.04	0.03	0.12	0.04	0.03	0.11	0.04	0.03	0.11
*Caregiver Age*		0.06	0.04	0.16	−0.01	0.00	<.0001	−0.01	0.00	<.0001	−0.01	0.00	<.0001	−0.01	0.00	<.0001
*Salary Imputation*	No (ref)															
	Yes	−0.01	0.00	<.0001	0.06	0.04	0.16	0.06	0.04	0.16	0.06	0.04	0.16	0.06	0.04	0.16
Disadvantage (CD)					−0.02	0.01	0.11				−0.01	0.02	0.55	−0.01	0.02	0.53
Collective Efficacy (CE)								0.04	0.02	0.01	0.04	0.02	0.03	0.03	0.02	0.05
CD*CE														0.00	0.02	0.86

**Table 6:** Variance Parameter Estimates for Change in Parental Caregiver Perceived Social Support.

	NeighborhoodLevelEst(SE)	CaregiverLevelEst(SE)	ICC^A^
**Null Model**	0.0001(0.0007)	0.1686(0.0047)***	0.1%
**Adjusted for compositional (i.e. caregiver) characteristics**	-	0.1657 (0.0046)***	-
**Adjusted for neighborhood concentrated disadvantage**	-	0.1657 (0.0046)***	-
**Adjusted for neighborhood collective efficacy**	-	0.1657 (0.0046)***	-

A Intra-class correlation coefficient, proportion of the unexplained variation in parental caregiver perceived social support attributable to the neighborhood level.

∼ p<.10,

* p<.05,

** p<.01,

*** p<.001.

## Discussion

Parental social support has been linked to positive parenting practices in the empirical literature [4], [6]. Additionally, studies have also linked neighborhood environment to parenting practices [14], [37]. However, little is known about whether and how the neighborhood environment impinges on or promotes parental social support. This study was conducted to address this knowledge-gap by exploring the influence of neighborhood structural and social processes on the *level* and *change* in parental caregiver perceived non-familial social support.

Our study found support for a relationship between neighborhood collective efficacy and *change* in parental perceived social support however we did not find support for the relationship between neighborhood collective efficacy and *level* of perceived social support. Discrepant findings between longitudinal and cross-sectional models are not uncommon in the literature [38]. Our findings seemed to suggest that neighborhood collective efficacy presented no immediate effect on a parental caregiver's level of perceived social support, but that the effects of collective efficacy could accumulate over time and have a lagged effect on the change in a caregiver's level of perceived social support. In order to explore this further, we tested the interaction between length of residence in a neighborhood and collective efficacy. The interaction term was not significant, suggesting that the beneficial effect of neighborhood collective efficacy on parental perceived social support is not stronger for longer term residents.

The observed one unit change in individual perceived social support for a .04 increase in collective efficacy is important when we consider that these effects are applied across the population to many caregivers. While this effect is small, it is “spread” across the population and community-wide interventions to promote neighborhood collective efficacy (in addition to individual level interventions to promote social support) may be a more efficient way to promote parental perceived social support across communities.

Other studies have found similar positive effects of living in neighborhoods with high collective efficacy for other outcomes [19], [27]. It is possible that parents living in neighborhoods characterized by high collective efficacy and collective support may be more likely to feel individually supported. This important finding persisted, even in models adjusting for neighborhood concentrated disadvantage. This suggests that living in a neighborhood characterized by collective action can induce the feeling of being supported in parental caregivers, irrespective of the socio-demographic resources available in a neighborhood.

This study is innovative from multiple perspectives. First, it fills an important gap in the literature by examining how support-inducing neighborhood processes, such as collective efficacy, may influence an important potential determinant of parenting—namely parental perceived social support. Second, this study is innovative in its use of a longitudinal approach to this question, given evidence that social support can fluctuate over the lifespan [29], [30]. Finally, unlike other studies of parenting and neighborhood context [13], our neighborhood variables were not derived from the participants themselves, but from an independent sample of residents from urban Chicago.

Despite these strengths, we acknowledge several limitations to this research. *First*, social support was measured via a self-report instrument which may not reflect the actual receipt of social support [39]. Despite this limitation, there is evidence that unless social support is perceived, it cannot be used [30]. *Second*, neighborhood characteristics utilized in this study were only measured at time point. It is possible that neighborhood characteristics changed over time. *Third*, it is possible that individual caregivers with high or low social support elected to move into particular neighborhoods. This may have influenced our findings away from the null. In order to address this limitation we implemented a rigorous adjustment for key caregiver level variables that could potentially explain selection into a particular neighborhood. In future studies, we will explore the use of analytic strategies such as instrumental variable analysis to address the issue of selection. *Fourth*, we recognize that administratively defined neighborhoods do not necessarily equate with “socially meaningful” neighborhoods. However every effort was made to ensure that the neighborhood clusters selected for this study approximated local neighborhoods and were internally homogenous on key census indicators. *Fifth*, parental perceived social support was only collected at two time points, Wave 1 and Wave 3, therefore we were restricted to a two wave longitudinal analysis. We recognize that this is a limitation of our study. It is possible that social support is erroneously measured as low at Wave 1 and high at Wave 2, suggesting there is improvement in parental perceived social support from Wave 1 to Wave 2. when in fact this is attributable to measurement error [40]. Despite this limitation, we believe the presentation of *both* cross sectional and longitudinal, albeit a two wave longitudinal model is a strength of this study. *Sixth*, while there are methods for constructing ecometric neighborhood measures of collective efficacy (e.g. systematic social observation) we utilized a measure constructed from the aggregated responses of individuals. While an ecometric measure would possibly capture the concept of collective efficacy as a collective characteristic, as suggested by Subramanian et al. (2002), these types of measures may fail to capture the “perceived social dynamism” that is an important component of a community's social capital. Furthermore these approaches are not yet well tested and are both time and cost intensive. *Seventh and final,* our study has limited generalizability to individuals living in neighborhood such as urban Chicago, and particularly to female caregivers as our sample was predominately female. Despite this limitation, the PHDCN was uniquely set up to allow us to move beyond traditional census and parental reported measures to capture neighborhood characteristics. It is also important to note that Chicago was chosen by PHDCN researchers because of the racial/ethnic and socioeconomic diversity seen across each neighborhood.

In future studies we intend to expand on this research and incorporate different measures of social support, by interviewing both the recipients and the providers of social support. We also hope to examine other types of social support, beyond emotional social support. For instance, instrumental social support may be particularly sensitive to neighborhood characteristics. Finally, we also intend to examine fluctuations in neighborhood attributes over time and relate these to changes in individual outcomes.

### Implications

Our finding that neighborhood environment matters for a parent's perception of feeling supported can inform the development of neighborhood level interventions for bolstering parental social support. An intervention designed to promote individual parental caregiver perceived social support would benefit from the inclusion of a community-wide effort to promote neighborhood collective efficacy. Collective efficacy stems from the shared belief by neighborhood individuals that they are capable of making a difference in their community and consequently are actively engaged in this process. This would not preclude providing interventions to enhance individual social support, however, a community-wide intervention would likely be a more efficient way to intervene and the potential benefit would be distributed across the population. Ultimately, an intervention that could promote parental social support would have great potential to improve parenting practices. [4], [41] and result in positive child outcomes.
